# Efficacy and safety of Tongmai Jiangtang capsule combined with conventional therapy in the treatment of diabetic peripheral neuropathy: a systematic review and meta-analysis

**DOI:** 10.3389/fneur.2023.1100327

**Published:** 2023-04-26

**Authors:** Lin-xi Sun, Yuan-yuan Li, Yan-ming Xie

**Affiliations:** ^1^Institute of Basic Research in Clinical Medicine, China Academy of Chinese Medical Sciences, Beijing, China; ^2^Yueyang Hospital of Integrated Traditional Chinese and Western Medicine, Shanghai University of Traditional Chinese Medicine, Shanghai, China

**Keywords:** diabetic peripheral neuropathy, Tongmai Jiangtang capsule, meta-analysis, system evaluation, traditional Chinese medicine

## Abstract

**Background:**

Recently, more and more Chinese patent drugs have been proved to be effective in the treatment of diabetic peripheral neuropathy (DPN). Tongmai Jiangtang capsule (TJC) is one of the representative ones. The present meta-analysis integrated data from several independent studies to determine the efficacy and safety of TJCs combined with routine hypoglycemic therapy for DPN patients, and to evaluate the quality of evidence.

**Methods:**

SinoMed, Cochrane Library, PubMed, EMBASE, Web of Science, CNKI, Wanfang, VIP databases and registers were searched for randomized controlled trials (RCTs) involving TJC treatment of DPN up to February 18, 2023. Two researchers independently used the Cochrane risk bias tool and comprehensive reporting criteria for Chinese medicine trials to evaluate the methodological quality and reporting quality of the qualified studies. RevMan5.4 was used for Meta-analysis and evidence evaluation, with scores determined for recommendations, evaluation, development and GRADE. The Cochrane Collaboration ROB tool was used to evaluate the quality of the literature. The results of Meta-analysis were represented by forest plots.

**Results:**

A total of 8 studies were included involving a total sample size of 656 cases. TJCs combined with conventional treatment (CT) could significantly accelerate myoelectricity graphic nerve conduction velocity, including that median nerve motor conduction velocity was faster than those of CT alone [mean difference (MD) = 5.20, 95% confidence interval (CI): 4.31–6.10, *P* < 0.00001], peroneal nerve motor conduction velocity was faster than those of CT alone (MD = 2.66, 95% CI: 1.63–3.68; *P* < 0.00001), median nerve sensory conduction velocity was faster than those of CT alone (MD = 3.06, 95% CI: 2.32–3.81, *P* < 0.00001), and peroneal nerve sensory conduction velocity was faster than those of CT alone (MD = 4.23, 95% CI: 3.30–5.16, *P* < 0.00001). The total efficiency of the TJCs + CT group was higher than that of the CT group (RR = 1.41, 95% CI: 1.28–1.56, *P* < 0.00001). The HbA1c after treatment in the TJCs + CT group was lower than that in the CT group (*P* < 0.05). No adverse drug reactions (ADRs) were reported in the combined TJCs or CT groups.

**Conclusions:**

TJCs combined with CT reduced the severity of DPN symptoms and no treatment-associated ADRs were reported. However, these results should be considered with caution because there was marked heterogeneity in the research data. Therefore, more stringent RCTs should be designed to validate the efficacy of TJCs in DPN patients.

**Systematic review registration:**

https://www.crd.york.ac.uk/PROSPERO/display_record.php?RecordID=264522, identifier: CRD42021264522.

## 1. Introduction

Diabetes mellitus is a chronic disease affecting the health and quality of life of a significant proportion of the population. One of the common debilitating complications of diabetes involves DPN, a symmetrical form of peripheral neuropathy. As diabetes develops, DPN begins as abnormal sensations but can progress to disabling neuropathic pain. According to national DPN screening conducted by the Diabetes Society of Chinese Medical Association, the prevalence of DPN in China was as high as 52.97% (1), with clinical symptoms mainly reported as abnormal sensations and movement of the extremity. The symptoms usually start from the toes and developed to the proximal extremities, showing a “glove and sock” distribution with muscle weakness often found in the late stage of the disease. About one third of patients with DPN will develop pain in their limbs, variously described by sufferers as burning or stabbing pain, electrical shock-like or cutting pain (2, 3). The lower extremity paresthesia caused by DPN is the primary cause of foot ulceration and even amputation, which greatly reduces the quality of life of patients and causes a huge social and economic burden.

At present, the pathogenesis of DPN has not been fully clarified, but it is generally known that the generation of nerve injury and pain is related to metabolic disorders, loss of nerve fibers, oxidative stress, inflammatory responses, genetic factors and the influence of lifestyle. Hu and Yang (4) proposed that DPN is caused by multiple factors associated with hyperglycemia including metabolic disorders, vascular damage, neurotrophic disorders, oxidative stress and genetic factors, resulting in activation of various metabolic pathways including the polyol pathway, hexosylamine pathway, protein kinase C pathway and also the formation of glycation end products. Xu et al. (5) suggested there were two main causes of the disease, namely, a lack of blood supply and pathophysiological changes in neurons or nerve fibers caused by high blood glucose. Oxidative stress is also key factor in many chronic complications of diabetes, including DPN. The current therapeutic goals of DPN are to relieve symptoms and prevent the development and progression of neuropathy. The various drug therapies used aim to normalize hyperglycemia, hypertension, and hyperlipidemia, to correct antioxidant stress, and to improve microcirculation and induce nerve repair. In this respect, traditional Chinese medicines and patent Chinese medicines have become important auxiliary methods for the clinical management of DPN.

TJCs are a kind of Chinese medicine to nourish Yin and clear heat, mainly comprised of *Radix Pseudostellariae, Radix Salviae Miltiorrhizae, Rhizoma Coptidis, Radix Astragali, Gynostemma pentaphylla, Rhizoma Atractylodis, Radix Scrophulariae, Hirudo, Fructus Malvae, Radix Puerariae*, and *Rhizoma Dioscoreae*. TJCs have the effect of nourishing the Yin and clearing heat and promoting blood circulation and can be used to treat diabetes caused by deficiency of qi and Yin and blood stasis of veins (6). Although clinical studies have reported that TJCs can be used adjuvant treatment in DPN, the evidence is relatively scattered and no relevant systematic review has presently been conducted to properly evaluate the quality of evidence. Therefore, the primary aim of this study was to address this problem by conducting a systematic review of the current literature. Toward this, literature that meets the quality standards was systematically and strictly screened to achieve an accurate evaluation of the effectiveness and safety of TJCs combined with Western medicine in the treatment of DPN. This study therefore provides an up-to-date reference for the clinical application of TJCs in DPN.

## 2. Methods

### 2.1. Study registration

This systematic review was registered in the PROSPERO (registration number: CRD42021264522), and referred to the “PRISMA 2020 Checklist” (http://www.prisma-statement.org) (7).

### 2.2. Search strategy

We searched the following eight databases and two register websites from their inception to February 18, 2023: PubMed, Embase, Cochrane Library, Web of Science, China National Knowledge Infrastructure, Chinese BioMedical Database, WanFang Database, VIP Database (VIP), https://clinicaltrials.gov/, and http://www.chictr.org.cn/. The following terms were searched in the abstract or title of the study: (Diabetic peripheral neuropathy OR peripheral neuropathy OR DPN) AND (Tongmai Jiangtang capsule OR Tongmai hypoglycemic capsule OR Tongmai Jiangtang) AND (clinical research OR clinical observation curative effect observation OR clinical efficacy OR clinical evaluation OR clinical trial OR RCT, etc), and the comprehensive search of subject words combined with free words is carried out based on the respective characteristics of the database. Take PubMed as an example, the detailed search strategy was shown in Supplementary Table 1. The detailed retrieval search strategies we have developed for each database are in Supplementary material. We also manually searched for studies that met the inclusion criteria from other sources not included in the above database, as well as for dissertations, conference papers, such as gray literature, and unpublished research results from relevant companies. Two researchers (Sun LX and Li YY) independently screened eligible studies, and identified inconsistencies by discussing them with other researchers. The studies were retrieved in Chinese and English languages.

### 2.3. Inclusion and exclusion criteria

The inclusion criteria were as follows: (1) only the RCTs was involved; (2) patients meeting diagnostic criteria for DPN: a diagnosis of diabetes (2), with arms and legs (at least in the double lower limbs) with persistent pain and/or sensory disturbance, abatement of vibratory sensation in both thumbs or at least one thumb, double side or side of the ankle reflex decreased to disappear, myoelectricity graphic double lower limbs nerve conduction velocity delay, median nerve, phil total nerve, motor nerve conduction velocity (MNCV) < 40 m/s, sensory nerve conduction velocity (SNCV) of sural nerve < 45 m/s, prolonged latency, decreased amplitude and other electrophysiological abnormalities (8, 9); (3) treatment groups were treated using oral TJCs combined with CT, according to the general treatment and etiological treatment methods in the Guidelines for the Prevention and Treatment of Type 2 Diabetes in China (10) (2020 edition) and control groups treated with CT (conventional drugs including mecobalamine, etc); Conventional drugs for the treatment of DPN refer to Table 1. (4) main outcome of median nerve motor nerve conduction velocity; secondary outcomes of peroneal nerve motor conduction velocity, median nerve sensory conduction velocity, and peroneal nerve sensory conduction velocity; and safety outcome of any ADR. The exclusion criteria were as follows: (1) clinical summative literature only; (2) studies in which data were incomplete or cannot be extracted, and republished literature; (3) the data had obvious errors; (4) incomparable objective outcome indicators.

**Table 1:** Conventional drugs for the treatment of DPN.

**Drug**	**NNT**	**AAN**	**NICE**	**EFNS**	**NeuPSIG IASP**
GABA analogs				First line	First line
Pregabalin	5.0	First line	First line		
Gabapentin	6.0		First line		
TCAs				First line	
Amitriptyline	1.3	Second line	First line		
Imipramine	2.2	Second line			First line
Desipramine	2.6				First line
SNRIs		Second line		First line	First line
Duloxetine	5.0		First line		
Venlafaxine	3.1				

AAN, American Academy of Neurology; EFNS, European Federation of Neurological Societies; IENFD, intraepidermal nerve fiber density; NeuPSIG IASP, Neuropathic Pain Special Interest Group of the International Association for the Study of Pain; NICE, National Institute for Health and Care Excellence; NNT, Number Needed to Treat for at least 50% pain relief.

Quoted from Khdour Maher (11).

### 2.4. Data extraction and risk of bias assessment

According to the standard information extraction table, the data was extracted independently by two researchers (Sun LX and Li YY). Throughout the process, differences were resolved through discussion or the participation of another researchers (Xie YM). The basic information extracted from articles included author's name, year of publication, type of study design, number of cases, gender, age, time from symptom onset to randomization, course of treatment, investigator group, treatment regimen, and outcome measures.

Two reviewers (Sun LX and Li YY) independently assessed the risk of bias in each study using the criteria outlined in the Cochrane Handbook for Systematic Reviews of Interventions (2019). Disagreements were judged by discussion or participation of another author (Xie YM). The quality of the literature was evaluated using the “bias risk assessment tool” recommended by the Cochrane Collaboration, which included the following domains: (1) randomization; (2) attrition bias; (3) allocation concealments; (4) blinding of participants and personnel; (5) blinding of outcome assessment; (6) integrity of outcome data; (7) selective reporting of study results; (8) other sources of bias. Each potential source of bias was described as “low,” “high,” and “unclear” (lack of relevant information or uncertainty of bias). The red part of the bias risk table represents high risk, green represents low risk, and yellow represents uncertain risk. Annotations were added to tables when information about the risk of bias and unpublished data sources were supplemented by contacting trial authors. We also included the risk of bias for each study, which may have influenced the outcome, in evaluating treatment effectiveness. Moreover, we used the Grading of Recommendations, Assessment, Development and Evaluation (GRADE) criteria to rank the quality of the evidence using the GRADE profiler (GRADEpro) software (12).

### 2.5. Data synthesis and analysis

RevMan 5.4 software (provided by the Cochrane Collaboration) was used to perform the meta-analysis and produce the risk of bias graph and risk of bias summary. For dichotomous data, risk ratios (RRs), Mantel Haenszel tests, and 95% CIs analysis were used. Continuous variables were expressed as MD, effect value and 95% CI. Statistical heterogeneity tests were performed with I^2^ values ranging from 0% to 100%: I^2^ ≤ 40%, were considered to have good statistical homogeneity, and the fixed effect model was adopted; 30% < I^2^ < 60%, mean moderate heterogeneity; 50% < I^2^ < 100%, show substantial statistical heterogeneity, and further analysis was undertaken to assess the reasons of the heterogeneity using subgroup and sensitivity analyses. And the random effects model was used in cases of obvious clinical heterogeneity. Meta-analysis was used to minimize potential clinical heterogeneity, and to identify the reasons for the heterogeneity, including curative effect evaluation standards, index, age, gender and conclusion group intervention measures, such as controlled drugs. For *P*-values of < 0.05, the difference was considered to be statistically significant. Meta-analysis results were presented as forest plots, and possible publication bias was analyzed by funnel plots. If the study was not suitable for meta-analysis, descriptive analysis was performed. If ≥10 articles were included in an outcome index, funnel plots were used to analyze whether publication bias existed.

## 3. Results

### 3.1. Search results and study characteristics

**Figure 1:**
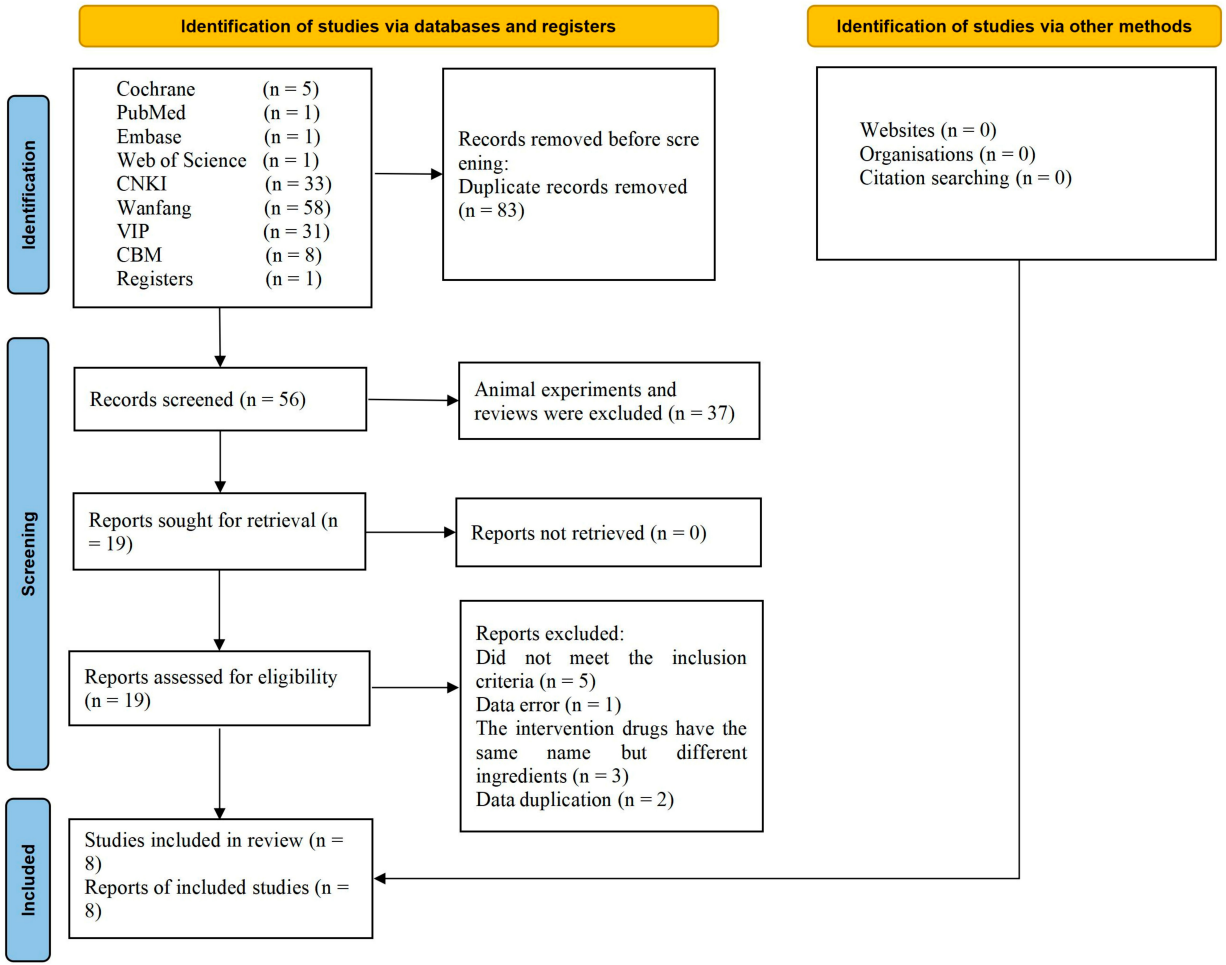
PRISMA flow diagram indicating the selection process for this meta-analysis.

A total of 139 studies were identified using the search strategy, including 5 articles from Cochrane library, 1 from PubMed, Embase and Web of Science, 33 from CNKI, 58 from Wanfang, 31 from VIP, 8 from CBM, 1 from register websites. Analysis of these for duplicate studies excluded a total of 83 studies. After reviewing the abstracts, another 37 studies were excluded for not meeting the inclusion criteria. Of the remaining 19 studies, 11 were excluded after full text review. Ultimately, 8 RCTs involving 656 cases were included (13–20). The flowchart of the screening process is presented in Figure 1. Two researchers independently extracted the data from the literature. There were 333 cases in the treatment group and 323 cases in the control group. All 8 RCTs were published in Chinese language between 2010 and 2015. The trial design for all 8 studies involved TJCs combined with CT (mecobalamin, etc.) vs. CT. Treatment courses ranged from 28 to 90 days. Five studies reported ADRs (15–19). The baseline characteristics were consistent across the studies. The detailed characteristics of the studies are presented in Table 2.

**Table 2:** Characteristics of included studies.

**Study id (location)**	**Cases**		**Gender (male/female)**	**Age**	**Course of disease**	**Intervening measure**	**Course of treatment**	**Treatment period**	**Outcome indicator**	**Randomization**	**Double Blinding**
	**T**	**C**	**T/C**	**T/C**	**T/C**	**T**	**C**				
Du (13) (China)	50	40	T: 28/22	T: 51.9 ± 10.1	T: 4.1 ± 1.8 years	TJCs 1.2g, tid + CT	Mecobalamine 1,000 ug, qd, iv drop	56 d	1234	MWD	N.R
			C: 25/15	C: 52.1 ± 9.8	C: 6.0 ± 2.1 years						
Mo (14) (China)	30	30	T: 16/14	T: 48–73 (58.81 ± 7.50)	T: 7–17 years	TJCs 1.2 g, tid + CT	α-lipoic acid 600 mg, qd, iv drop + Mecobalamine 500 ug, qd, iv	28 d	12345	MWD	N.R
			C: 14/16	C: 47–73 (59.12 ± 8.03)	C: 7–16 years						
Kong (15) (China)	60	60	T: 34/26	T: 53–81 (67.9 ± 5.8)	NA	TJCs1.2 g, tid + CT	Mecobalamine 500 ug tid, po	60 d	123457	MWD	N.R
			C: 32/28	C: 53–81 (67.3 ± 4.6)							
Xu and Chen (16) (China)	50	50	T: 24/24 (out of 2 cases)	T: 44–70 (55.0 ± 3.6)	T: 9 months−22 years (5.8 ± 4.6 years)	TJCs 0.8 g, tid + CT	Mecobalamine 500 ug, tid, po	60 d	2357	Random number table	N.R
			C: 22/25 (out of 3 cases)	C: 42–68 (53.3 ± 4.8)	C: 7 months−19 years (6.7 ± 3.4 years)						
Chen et al. (19) (China)	30	30	T: 17/13	T: 37–81	T: 19 cases ≥12 months, 11 cases < 12 months	TJCs 1.2 g, tid + CT	Insulin glargine 8 U, qd, ih + Acarbose 50 mg, tid + Mecobalamine 0.5 mg, tid	90 d	567	MWD	N.R
			C: 16/14	C: 36–80	C: 18 cases ≥12 months, 12 cases < 12 months						
Zhang et al. (17) (China)	53	53	T: 32/21	T: 35–70	T: 12–36 months	TJCs 1.2 g, tid + CT	Mecobalamine 500 ug tid po	90 d	123457	MWD	N.R
			C: 31/22	C: 33–70	C: 10–32 months						
Wang (18) (China)	30	30	T: 16/14	T: 50–70	T: 10 months−6 years	TJCs 1.2 g, tid + original hypoglycemic regimen (details unknown)	Mecobalamine 500 ug, tid, po + original hypoglycemic regimen (details unknown)	90 d	123457	MWD	N.R
			C: 17/13	C: 49–71	C: 9 months−6 years						
Hao et al. (20) (China)	30	30	T:17/13	T: 29–78 (52.12 ± 3.24)	T: 2–10 (4.84 ± 2.22) years	TJCs 1.2 g, tid + CT	Vitamin B1 (100 mg) and B12 (500 μg), im, qd	60 d	5	MWD	N.R
			C:14/16	C: 28–77 (52.36 ± 2.54)	C: 2–11 (4.78 ± 2.15) years						

T, experimental group; C, control group; NA, no available; The outcomes are Median nerve motor conduction velocity, Peroneal nerve motor conduction velocity, Median nerve sensory conduction velocity, Peroneal sensory conduction velocity, total effective rate, glycosylated hemoglobin and adverse reactions, that are denoted by “1” “2” “3” “4” “5” “6” and “7” respectively; MWD, mentioned without description; N.R, not reported.

### 3.2. Methodological quality assessment

**Figure 2:**
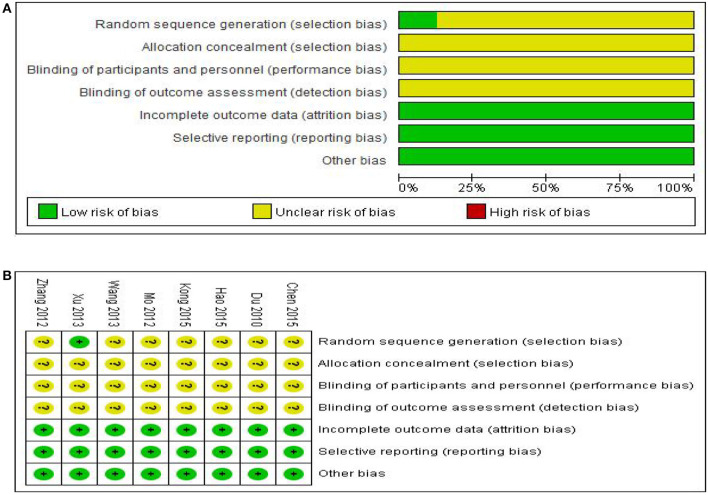
Assessment of risk of bias in the eight trials. **(A)** Risk bias results proportional chart, **(B)** Summary chart of risk bias results.

Risk assessment of bias for the seven trials is shown in Figure 2. All studies were randomly grouped, one used a random number table, and no specific randomization method was reported for the remaining trials. None of the eight studies provided blind information and assignment concealment, and it was not clear whether the randomly assigned investigator and assignment concealment investigator were third-party personnel. As a result, the studies were rated as having ambiguous risks. One study reported in detail the number and reasons for exclusions, and seven studies reported results based on preset outcome indicators; thus all were rated as low risk in terms of outcome data completeness and selective reporting. All studies showed no significant other biases and were therefore rated as low risk.

We should also note that we attempted to contact the published author of each study by phone or email to ask for details of the trial to assess the methodological quality in greater detail but did not receive any responses. It is therefore unclear whether the included studies used strict operating procedures such as allocation, concealment and blinding. Given the overall methodological quality of the included studies was considered poor, the results of this study must be viewed in this context.

## 4. Primary outcome

### 4.1. Median nerve motor conduction velocity

**Figure 3:**
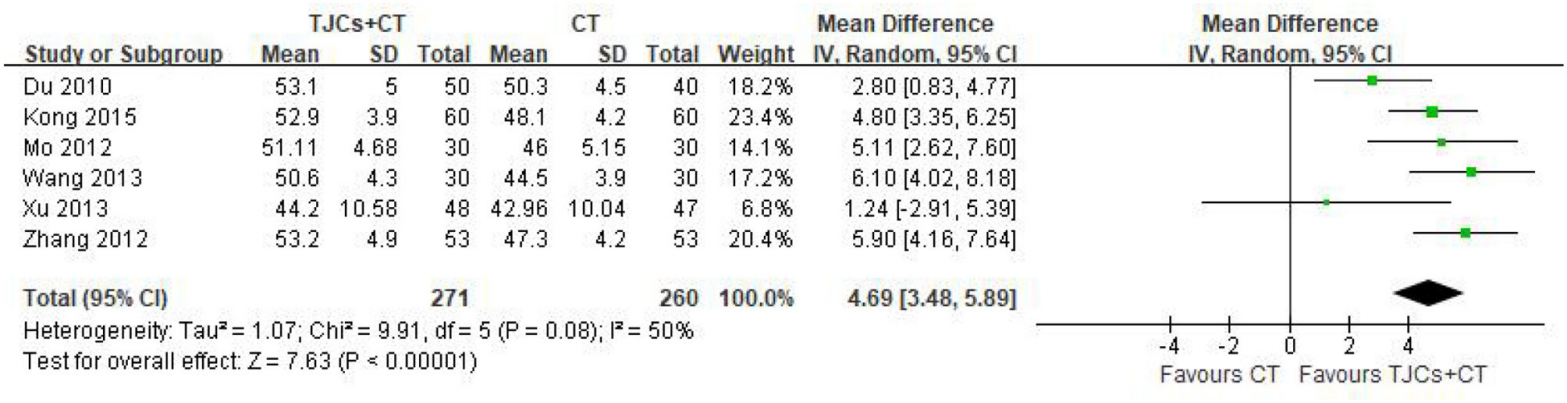
Forest plot of Median nerve MNCV (m/s).

Six studies involving 531 cases reported the median nerve median nerve motor conduction velocity (MNCV) (13–18). The forest plot showed significant differences between the TJCs + CT groups and the CT groups (MD = 4.69, 95% CI: 3.48–5.89, *P* < 0.00001; Figure 3), and the heterogeneity was high (*P* = 0.08, I^2^ = 50%). The random effect model was selected to analyze the data according to heterogeneity. The results suggested that the MNCV of the median nerve was faster in the TJCs + CT group than in the CT group after treatment.

## 5. Secondary outcomes

### 5.1. Peroneal nerve motor conduction velocity (MNCV)

**Figure 4:**
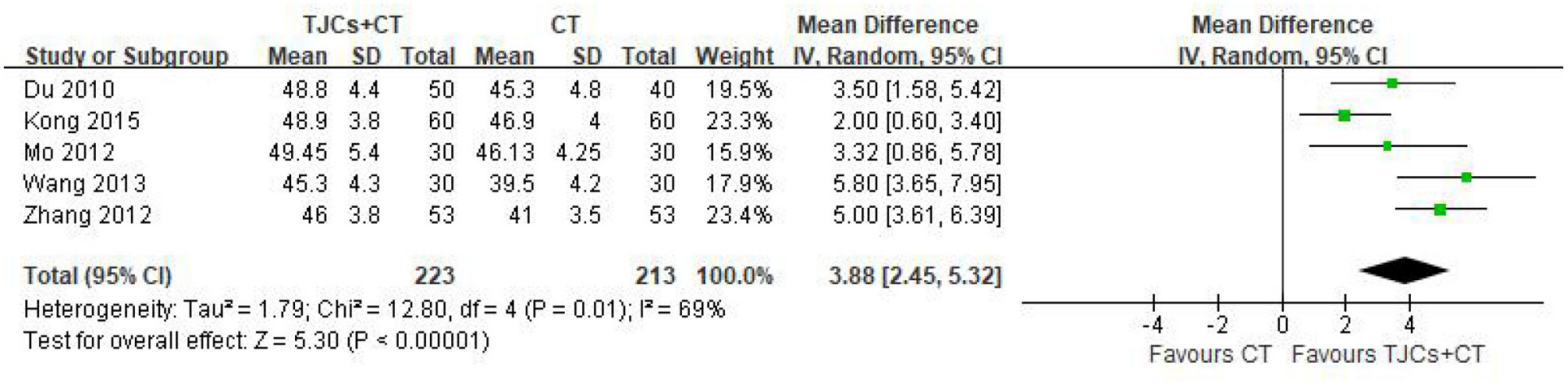
Forest plot of Peroneal nerve MNCV (m/s).

Five studies involving 436 cases reported the peroneal nerve MNCV (13–18). The forest plot (Figure 4) showed significant differences between the TJCs + CT groups and the CT groups (MD = 3.88, 95% CI: 2.45–5.32, *P* < 0.00001). Moreover, it has high heterogeneity (*P* = 0.01, I^2^ = 69%). The random effects model was used to analyze the data according to heterogeneity. The results suggested that the MNCV of the peroneal nerve was faster in the TJCs + CT group than in the CT group after treatment.

### 5.2. Median nerve sensory conduction velocity (SNCV)

**Figure 5:**
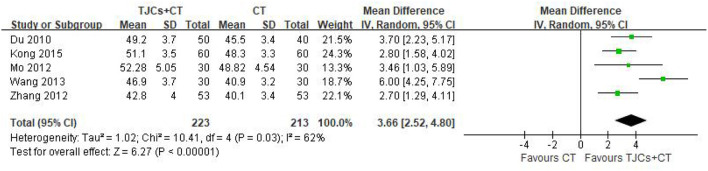
Forest plot of Median nerve SNCV (m/s).

Five studies involving 436 cases reported the median nerve SNCV (13–18). The forest plot (Figure 5) showed significant differences between the TJCs + CT groups and the CT groups (MD = 3.66, 95% CI: 2.52–4.80, *P* < 0.00001). Moreover, it has high heterogeneity (*P* = 0.03, I^2^ = 62%). The random effects model was used to analyze the data according to heterogeneity. The results suggested that the SNCV of the median nerve was faster in the TJCs + CT group than in the CT group after treatment.

### 5.3. Peroneal sensory conduction velocity (SNCV)

**Figure 6:**
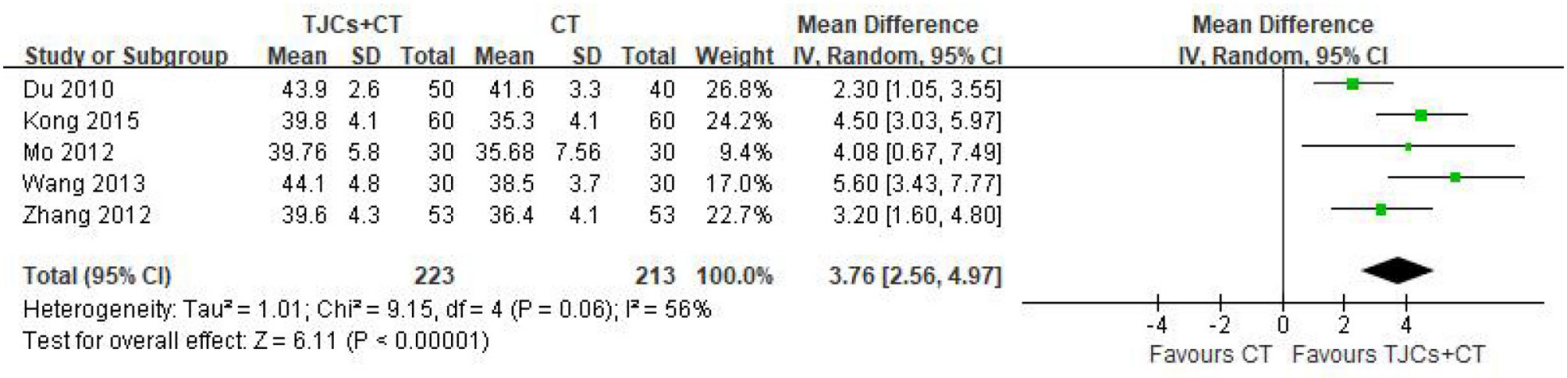
Forest plot of Peroneal nerve SNCV (m/s).

Five studies involving 436 cases reported the peroneal nerve SNCV (13–18). The forest plot (Figure 6) showed significant differences between the TJCs + CT groups and the CT groups (MD = 3.76, 95% CI: 2.56–4.97, *P* < 0.00001). Moreover, it has high heterogeneity (*P* = 0.06, I^2^ = 56%). The random effects model was used to analyze the data according to heterogeneity. The results suggested that the SNCV of the peroneal nerve was faster in the TJCs + CT group than in the CT group after treatment.

### 5.4. Total effective rate

**Figure 7:**
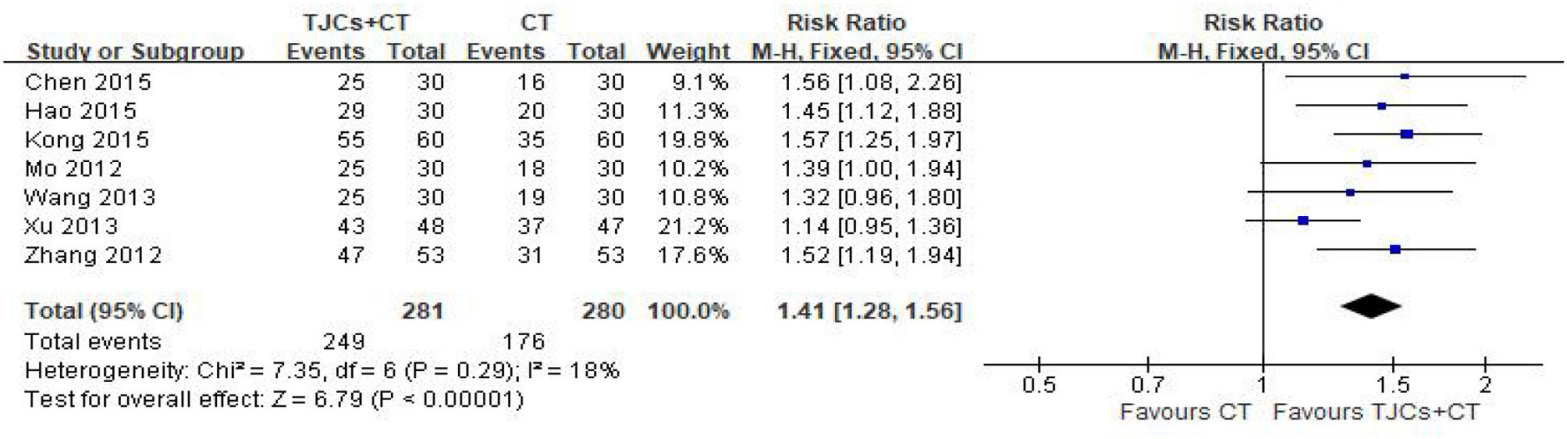
Forest plot of total effective rate.

Seven studies involving 561 cases reported the total effective rate (14–20). The forest plot (Figure 7) showed significant differences between the TJCs + CT groups and the CT groups (RR = 1.41, 95% CI: 1.28–1.56, *P* < 0.00001). Moreover, it has low heterogeneity (*P* = 0.29, I^2^ = 18%). The fixed effects model was used to analyze the data according to heterogeneity. The results suggested that the total efficiency of the TJCs + CT group was higher than that of the CT group.

### 5.5. Glycosylated hemoglobin (HbA1c)

One study involving 60 cases reported HbA1c (19). The HbA1c after treatment was lower than that before treatment in both groups, and the HbA1c after treatment in the TJCs + CT group was lower than that in the CT group (*P* < 0.05) (Table 3).

**Table 3:** HbA1c of TJCs + CT vs. CT **(%)**.

**Chen et al. (20)**	**TJCs + CT (*n* = 30)**	**CT (*n* = 30)**
Pre-treatment	8.16 ± 1.04	7.93 ± 1.17
Post-treatment	6.28 ± 0.12^*#^	7.02 ± 0.57^*#^

Compared with the same index before treatment in the same group, ^*^P < 0.05; compared with the same index after treatment in the control group, ^#^P < 0.05.

### 5.6. Safety analysis: ADRs

5 literatures involving 446 cases (15–19) were included to report the safety of medication, and all the experimental groups were TJCs combined with CT. There were no ADRs in the experimental group and control group in the 5 clinical observations.

### 5.7. Sensitivity analysis

After deleting individual studies one by one we observed the changes of I^2^ (Table 4), conducted sensitivity analysis, and then explored the potential sources of heterogeneity. With regard to median nerve MNCV, the heterogeneity was the lowest (*P* = 0.27, *I*^2^ = 22%) after removal of the study of Du, which may be related to the inconsistent sample sizes between the experimental and control groups in this study. For peroneal nerve MNCV, the heterogeneity was the lowest (*P* = 0.39, I^2^ = 0%) after removal of the study of Wang Qing and Zhang Aiqi, which may be related to the fact that the original hypoglycemic regimen of the patients in the experimental group and control group combined in Wang Qing's study was not detailed. Similarly, the heterogeneity of median nerve SNCV was lowest (*P* = 0.74, I^2^ = 0%) after removal of the Wang study, again likely related to specificity issues concerning the hypoglycemic regimen in the experimental and control groups. For peroneal nerve SNCV, the heterogeneity was the lowest (*P* = 0.35, I^2^ = 8%) after removal of the study of Du, which again may be related to the inconsistent sample sizes between the experimental and control groups. The heterogeneity results did change to some extent.

**Table 4:** Heterogeneity and MD in sensitivity analysis of outcome indicators.

**Exclusion study**	**Median nerve MNCV**		**Peroneal nerve MNCV**		**Median nerve SNCV**		**Peroneal nerve SNCV**	
	**Pooled MD (95% CI)**	**Heterogeneity value;** ***I**^2^*	**Pooled MD (95% CI)**	**Heterogeneity value;** ***I**^2^*	**Pooled MD (95% CI)**	**Heterogeneity value;** ***I**^2^*	**Pooled MD (95% CI)**	**Heterogeneity value;** ***I**^2^*
Du (13) (China)	5.20 (4.31, 6.10)	0.27; 22%	3.99 (2.17, 5.81)	0.005; 76%	3.68 (2.17, 5.19)	0.02; 71%	4.23 (3.30, 5.16)	0.35; 8%
Mo (14) (China)	4.58 (3.15, 6.01)	0.04; 59%	4.00 (2.28, 5.72)	0.005; 76%	3.71 (2.36, 5.06)	0.02; 71%	3.75 (2.38, 5.12)	0.03; 67%
Kong (15) (China)	4.58 (2.97, 6.20)	0.04; 60%	4.56 (3.64, 5.49)	0.28; 21%	3.94 (2.51, 5.38)	0.04; 65%	3.55 (2.10, 5.00)	0.07; 57%
Xu and Chen (16) (China)	4.94 (4.11, 5.76)	0.14; 43%	/	/	/	/	/	/
Chen et al. (19) (China)	/	/	/	/	/	/	/	/
Zhang et al. (17) (China)	4.49 (3.57, 5.40)	0.09; 49%	3.54 (1.91, 5.18)	0.04; 65%	3.94 (2.54, 5.35)	0.03; 66%	3.98 (2.37, 5.59)	0.03; 66%
Wang (18) (China)	4.38 (3.04, 5.73)	0.09; 51%	3.47 (1.98, 4.95)	0.03; 66%	3.06 (2.32, 3.81)	0.74; 0%	3.27 (2.47, 4.06)	0.16; 43%

### 5.8. Evaluation of publication bias

Less than 10 studies were included for each outcome index, which did not meet the requirements for making a funnel plot. Hence there was no need to conduct publication bias analysis for the main outcome index.

### 5.9. Grading quality of evidence

The GRADEpro (www.gradepro.org) has been used to conduct evaluation on the convincing level of evidence and strength of recommendations for the involved outcomes. Initially, RCTs were considered to have high confidence, and cohort studies low confidence as for the estimate of effect. Factors which may have decreased the level of confidence level included inconsistency, limitations, imprecision, indirectness, as well as publication bias. As only RCTs could be included in the review, the reasons for the update (large effects, dose-response relationships and plausible confounders) do not apply. We present the results of GRADE analysis in Table 5.

**Table 5:** GRADE quality grading evaluation.

**Certainty assessment**							**No. of patients**		**Absolute (95% CI)**	**Certainty**
**Outcomes**	**Study design**	**Risk of bias**	**Inconsistency**	**Indirectness**	**Imprecision**	**Publication bias**	**T**	**C**		
Median nerve MNCV	RCTs	Very serious^a^	Not serious	Not serious	Not serious	Strongly suspected^bc^	271	260	MD 4.69 higher (3.48 higher to 5.89 higher)	⊕○○○ Very low
Peroneal nerve MNCV	RCTs	Very serious^a^	Not serious	Not serious	Not serious	Strongly suspected^bc^	223	213	MD 3.88 higher (2.45 higher to 5.32 higher)	⊕○○○ Very low
Median nerve SNCV	RCTs	Very serious^a^	Not serious	Not serious	Serious	Strongly suspected^bc^	223	213	MD 3.66 higher (2.52 higher to 4.8 higher)	⊕○○○ Very low
Peroneal nerve SNCV	RCTs	Very serious^a^	Not serious	Not serious	Serious	Strongly suspected^bc^	223	213	MD 3.54 higher (2.8 higher to 4.29 higher)	⊕○○○ Very low

CI, confidence interval; RR, relative risks.

^a^Poor methodology, including the method of randomization and blinding; ^b^small sample sizes; ^c^All results were positive.

## 6. Sample size evaluation

Trial sequential analysis (TSA) for systematic review or meta-analysis sample size estimation overcomes the shortcomings of classical systematic review or meta-analysis (21). First, when the number of cases included in a meta-analysis does not reach a sufficient sample size, the application of TSA minimizes the false positive results due to random errors results. Second, meta-analysis is a retrospective study, and the required information size (RIS) obtained by TSA refers to the number of cases needed to obtain statistically significant differences in meta-analysis, which is the sample size of meta-analysis. The sample size required for meta-analysis is generally considered to be no less than that required for a well-designed, statistically robust, single randomized controlled trial. Again, meta-analysis aims to find evidence of the efficacy of medical interventions as early as possible, and TSA provides a termination criterion for clinical trials by estimating RIS, thus avoiding waste of research and medical resources.

**Figure 8:**
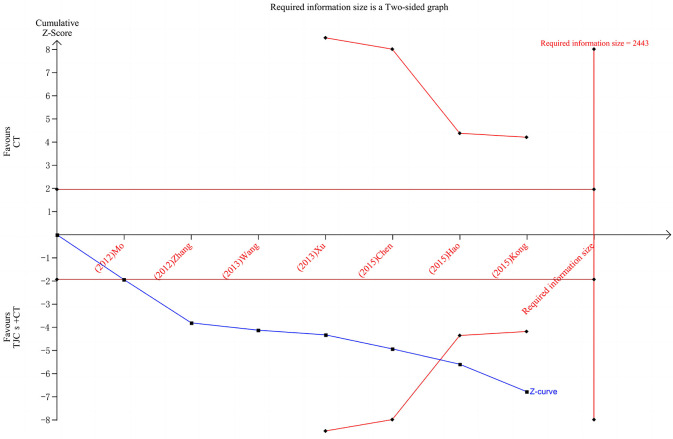
TSA of the total effective rate of TJCs + CT in the treatment of DPN. Red curve, TSA boundaries; Red vertical line, required information size; Blue curve, Cumulative Z-values; Black dashed line, Traditional cut-off values.

TSA was used to estimate the sample size of the seven studies reporting the total effective rate. The results of TSA analysis (Figure 8) showed that the cumulative Z value after the first study crossed the traditional cut-off curve (Z = 1.96) and crossed the TSA cut-off after the 6th study, indicating that although the cumulative information size did not reach the desired value, no more trials were needed to obtain a positive conclusion in advance (22).

## 7. Discussion

As far as we know, this study is the first systematic review of the effect of TJCs combined with conventional therapy in the treatment of DPN, and the quality of the evidence was assessed using GRADEpro. The TSA was used to calculate the sample size to estimate the effect more conservatively. The primary objective findings were that, compared with CT alone, the MNCV and SNCV of median and peroneal nerves were faster when Tongmai Jiangtang capsules were combined with CT, suggesting that TJCs play an active role in the treatment of DPN. However, the heterogeneity among these studies was high. In addition, the quality of research methodology was low, perhaps because of the disjointed comparisons in research designs between different selections of Western medicines and dosage forms. In addition, most of the studies did not mention specific randomization methods, consequently reducing the baseline comparability between groups, which may lead to selective bias (23). Furthermore, the studies did not mention the implementation of blinding methods, which did not exclude the implementation and measurement bias resulting in increased inter-group heterogeneity. Considering the possible bias and large heterogeneity, the effectiveness of TJCs in the treatment of DPN needs to be confirmed by more evidence-based research.

Chinese medicine theory of diabetes argues that the disease does not heal rapidly, which consumes body fluid, and this injury consumes qi. Qi deficiency affects the smooth flow of blood, resulting in blood stasis. Qi and blood cannot run to the end of the limbs, tendons and veins. In short, the pathogenesis of the disease is deficiency of Qi, blood, Yin and Yang, which causes blood stasis, and blood stasis is basis for inducing and accelerating the development of DPN. Thus, DPN marks a basic deficiency involving qi, Yin, and associated blood stasis (24). Indeed, cluster analysis by Pan et al. (25) showed that the pathogenesis of the main syndrome of DPN was mainly characterized by blood stasis, which was related to the spleen and kidney, and mainly manifested as deficiency of qi and Yang in the spleen and kidney. Notably, the patients diet can damage the spleen and stomach, lung and kidney, and the resulting qi deficiency can obstruct blood and promote blood stasis. Protracted spleen and kidney qi deficiency will eventually involve yuan Yang, cold appearance, and thus cause Yang deficiency of the spleen and kidney. Therefore, invigorating qi and nourishing Yin to promote blood circulation and remove blood stasis is one of the important measures for treating DPN.

The components of TJCs are diverse, with each component promoting different mechanisms of action to help treat diabetes and reverse the underlying causes of DPN. In terms of drug composition, *Radix scrophulariae* and *Radix astragalus* can replenish qi and nourish Yin, acting to both lower and regulate blood sugar. Here they act to promote blood circulation, improve microcirculation, improve the hemorheology index and improve blood supply to microvessels to help nurture nerves (26, 27). With *Rhizoma coptidis, Radix pseudostellariae, Hirudo*, and *Radix puerariae* as matching herbs, TJCs have the effects of supplementing qi, promoting blood circulation and producing fluid. *Salvia miltiorrhiza* and *Hirudo* can also inhibit platelet aggregation, reduce blood viscosity, dilate blood vessels, improve microcirculation, thereby increasing blood supply, oxygenation and nutrition in peripheral limb nerves, promoting the repair of injured peripheral nerves, and improving motor nerve conduction speed (18, 28). *Rhizoma Dioscoreae* and *Gyundum pentaphyllum* have the effects of promoting blood circulation and removing blood stasis, strengthening kidneys and nourishing essence, strengthening the spleen and nourishing the lung, regulating lipids and lowering blood sugar (14, 19). *Fructus Malvae* has a diuretic effect and assisted by *Rhizoma Atractylodis* can dispel wind and stasis dredge collaterals, disperse cold and promote dehumidification (29). Lastly, *Radix Puerariae* improves insulin resistance in patients with type 2 diabetes through a multiplicity of effects, protecting vascular endothelium and endothelial function, improving the hemorheology index, microcirculation and the central nervous function of diabetic patients.

In summary, the combined use of various drugs found in TJCs can promote Yin, clear heat, invigorate qi, invigorate the spleen, remove dampness, and promote blood circulation. The resulting effects treat both the symptoms and root causes of DPN. Moreover, these effects are in line with TCM's drug selection for differentiation of DPN syndrome. This also reflects the advantages of this treatment approach and underscores the ability of TCM to shows synergistic effects when combined with CT.

Along with clinical use, the activity and mechanism of Chinese herbal medicine components including TJCs has also been evaluated in experimental model systems. Investigations by Zhang et al. (30) showed that TJCs could inhibit the expression of inflammatory factors such as tumor necrosis factor (TNF-α) and interleukin 6 (IL-6). Moreover, they showed that TJCs directly act on Schwann cells (SC) in peripheral nerves, significantly inhibiting SC apoptosis induced by advanced glycation end products (AGES) by down-regulating TNF-α and IL-6 mRNA and protein expression. Interestingly, TJCs produced no significant effects on the expression of brain-derived nerve growth factor (BDNF) and nerve growth factor (NGF), proposing that the effects of TJCs in DPN involve a protective role through delaying SC apoptosis. The authors suggested that the mechanism of action of TJCs on SC may be closely related to inflammation theory of the pathogenesis of diabetes. Notably, this study confirms that TJCs not only act on microcirculation but also elicit direct effects on nerve cells.

A network-based pharmacological mechanism study of *Radix scrophulariae* in the treatment of diabetic foot disease found that the main active component was β-sitosterol, followed by salicinol, radix scrophulariae glycosides, and glutosterol in that order. Previous studies have shown that β-sitosterol is a phytosterol with a structure similar to cholesterol. Notably, in a rat model of diabetes induced by a high-fat and sucrose diet, β-sitosterol was shown to attenuate insulin resistance in adipose tissue by modulating the IRS-1/Akt signaling pathway (31). *Radix scrophulariae* can also induce regulatory T cells in humans through increasing the expression of Foxp3, transforming growth factor-β and IL-10 mRNA in resting peripheral blood mononuclear cells (27), therefore highlighting its potential as an anti-inflammatory agent. Furthermore, *Radix Astragali* was shown to significantly improved blood glucose levels in diabetic mice, and also to significantly reduce renal cell injury and glomerular cell apoptosis. This occurred through reductions in the levels of inflammatory response by a mechanism related to activation of the protease-activated receptor 2 (PAR2) signaling pathway (32). Other work showed the key signaling pathways targeted by the combination of *Rhizoma Atractylodis* and *Radix scrophulariae* included the regulation of lipolysis in adipocytes, PPAR signaling pathway, insulin resistance, and the AMPK signaling pathway. The identity of these signaling pathways strongly suggests that the mechanism of DPN treatment may be also related to the regulation of lipolysis (33). The combination of *Radix Astragali* with *Radix Puerariae* can also lower blood glucose with optimal effects, with evidence provided to suggest its hypoglycemic mechanism of action may be related to regulating adipokines and mediating AMPK/GLUT-4 transactivation (34).

The pharmacodynamic mechanism of TCM is now being clarified through the combination of ancient theory and modern technology. This approach provides strong guidance for the application of multi-target Chinese patent medicines in the treatment of clinical syndromes such DPN. In this study, we conducted a systematic evaluation based on the reported clinical literature. This provided the conclusion that the efficacy of clinical combination with TJCs in the treatment of DPN was better than that of symptomatic treatment alone. Regardless, such combinations still needs to be applied in accordance with their indications and functional specifications. However, among the included studies, five of eight documented safety results with notably no ADRs reported in either the experimental or control groups. Importantly this indicates that TJCs have no obvious toxicity and side effects, further suggesting that the combination TJCs with CT is safe and suitable for prolonged treatment use. Nevertheless, considering that the ADRs in the drug instructions are not clear, more clinical trials are needed to provide evidence to support the safety evaluation of TJCs in order to achieve clinical standardization and rational safe drug use.

The limitations and prospects of this study are as follows: (1) there are limitations with respect to region and race given the study sites of all evaluated trials were limited to China, and the subject race was not specified; thus the effect of “people and places” was ignored; (2) the research involved different combinations of drug choice, dosage and form. Thus, drug usage was not necessarily the same, particularly for the Western treatments where different medicines and administration methods were used, e.g., insulin and glucosaminic acid-cobalt chelate (35). (3) The included studies did not specifically mention the occurrence of severe syndromes or complications for invalid cases; (4) most studies did not carry out TCM syndrome differentiation for diabetic peripheral neuropathy, and ignored the efficacy of TJC in treating qi and Yin deficiency and vein stasis syndrome; (5) as the quality of the methodology was generally not high, which affects the reliability of the conclusions, clinicians should carefully consider the results of this study accordingly (36). Therefore, it is suggested that in clinical study design, disease differentiation and syndrome differentiation should be combined according to drug instructions to make clinical drug use more accurate and reasonable.

The implications based on the results of this study are as follows: (1) combining TJCs with CT in the treatment of DPN has a good overall effect. It is suggested that TJCs can be added to the clinical treatment of DPN, TJCs can be added on the basis of CT, while paying attention to ADRs; (2) the generally low methodological quality highlights the need for more rigorous research involving larger samples sizes with studies conducted properly in multicenter settings. In particular, there should be strict implementation of randomized controlled trial quality standards to study the effects of TJCs in the treatment of DPN in order to improve the quality levels of evidence for both efficacy and safety (37, 38). This will provide strong evidence and improved guidance for using TJCs in clinical practice; (3) currently, there is still a lack of uniform clinical norms for the outcome indicators of DPN treatment with Chinese patent medicine, and the total clinical response rate as an outcome indicator has not been internationally recognized (39). To increase the comparability between trials, it is suggested that future studies should first consider MNCV and SNCV as the main outcome indicators for efficacy evaluation; (4) proprietary Chinese medicines follow the ideas of TCM syndrome differentiation and treatment. On this basis, future research should be conducted accordingly with a focus on qi and Yin deficiency, and vein stasis resistance. This will help rule out deficiencies in theoretical guidance that might otherwise lead to poor curative effect of proprietary Chinese medicine in the clinic; (5) in the evaluation of outcome indicators, attention should be paid to exploring the long-term therapeutic effect of TJCs, particularly to increase the examination of objective indicators during follow-up; (6) it is suggested that researchers should pay more attention to the quality of future randomized controlled trials, referring to the standard protocol items of the SPIRIT statement (40). Such research should be carried out in strict accordance with this scheme, with trials featuring a normative design, scheme registration and strengthening of ADR monitoring. The reporting of the results should refer to the CONSORT Statement (Consolidated Standards of Reporting Trials, CONSORT) (41) to further evaluate the clinical efficacy and safety of TJCs.

Finally, we should note that a random effects model was used for meta-combination of four outcome data, mainly because the RCTs of each study did not have unified control group of hypoglycemic drugs, and the quality control of the RCTs was not sufficiently strict. Future RCTs should consider stratifying patients by key factors as much as possible, for example, by age and disease course, etc. In addition, more RCTs should be registered on standardized clinical trial platforms to describe the testing process in detail, so that studies can be more accurate and rigorous.

## 8. Conclusion

Our Meta-analysis investigated the efficacy and safety of TJCs combined with CT in the treatment of DPN, providing evidence to support the use of combination therapy. However, since most of the included studies were of low quality and the analysis results showed high heterogeneity, there is limited evidence to support this conclusion. This deficiency needs to be addressed in future high-quality, well-designed, multi-center RCTs. Moreover, future clinical studies should focus on improving objectivity in outcome measurements and methodological quality by adopting a rigorous experimental design. This research should strictly follow unified registration procedures and standards to provide higher levels of evidence for clinical applications of TJCs.

## Data availability statement

The original contributions presented in the study are included in the article/Supplementary material, further inquiries can be directed to the corresponding author.

## Author contributions

Concept design: Y-mX and L-xS. Literature screening, data collection, and integration inspection: L-xS and Y-yL. Manuscript writing: L-xS. Data analysis and interpretation and final review: All authors. All authors contributed to the final draft of the article.
