# Supplementary material for: Efficacy and safety of Tongmai Jiangtang capsule combined with conventional therapy in the treatment of diabetic peripheral neuropathy: a systematic review and meta-analysis
Source: Front Neurol. 2023 Apr 26;14:1100327. doi: 10.3389/fneur.2023.1100327 (PMC10171201; doi:10.3389/fneur.2023.1100327)
Supplement: Supplementary file 1 [file Data_Sheet_1.docx]

**Table 1**

The search strategy for Cochrane Library

| Order | Strategy |
| --- | --- |
| #1 | diabetic peripheral neuropathy OR peripheral neuropathy OR diabetic polyneuropathy OR DPN |
| #2 | Tongmai Jiangtang OR Tongmai hypoglycemic capsule |
| #3 | random OR randomization OR randomized OR randomised OR randomly |
| #4 | #1 AND #2 AND #3 in Trials |

**Table 2**

The search strategy for Pubmed

| ((("diabete"[All Fields] OR "diabetes mellitus"[MeSH Terms] OR ("diabetes"[All Fields] AND "mellitus"[All Fields]) OR "diabetes mellitus"[All Fields] OR "diabetes"[All Fields] OR "diabetes insipidus"[MeSH Terms] OR ("diabetes"[All Fields] AND "insipidus"[All Fields]) OR "diabetes insipidus"[All Fields] OR "diabetic"[All Fields] OR "diabetics"[All Fields] OR "diabets"[All Fields]) AND ("peripheral nervous system diseases"[MeSH Terms] OR ("peripheral"[All Fields] AND "nervous"[All Fields] AND "system"[All Fields] AND "diseases"[All Fields]) OR "peripheral nervous system diseases"[All Fields] OR ("peripheral"[All Fields] AND "neuropathy"[All Fields]) OR "peripheral neuropathy"[All Fields])) OR ("peripheral nervous system diseases"[MeSH Terms] OR ("peripheral"[All Fields] AND "nervous"[All Fields] AND "system"[All Fields] AND "diseases"[All Fields]) OR "peripheral nervous system diseases"[All Fields] OR ("peripheral"[All Fields] AND "neuropathy"[All Fields]) OR "peripheral neuropathy"[All Fields]) OR ("diabetic neuropathies"[MeSH Terms] OR ("diabetic"[All Fields] AND "neuropathies"[All Fields]) OR "diabetic neuropathies"[All Fields] OR ("diabetic"[All Fields] AND "polyneuropathy"[All Fields]) OR "diabetic polyneuropathy"[All Fields]) OR "DPN"[All Fields]) AND (("Tongmai"[All Fields] AND "Jiangtang"[All Fields]) OR ("Tongmai"[All Fields] AND ("hypoglycaemics"[All Fields] OR "hypoglycemic agents"[Pharmacological Action] OR "hypoglycemic agents"[MeSH Terms] OR ("hypoglycemic"[All Fields] AND "agents"[All Fields]) OR "hypoglycemic agents"[All Fields] OR "hypoglycaemic"[All Fields] OR "hypoglycemic"[All Fields] OR "hypoglycemics"[All Fields]) AND ("capsule s"[All Fields] OR "capsules"[MeSH Terms] OR "capsules"[All Fields] OR "capsule"[All Fields]))) |
| --- |

**Table 3**

The search strategy for EMBASE

| Order | Strategy |
| --- | --- |
| #14 | #4 AND #7AND #13 |
| #13 | #8 OR #9 OR #10 OR #11 OR #12 |
| #12 | randomly' |
| #11 | randomised' |
| #10 | randomized' |
| #9 | randomization' |
| #8 | random' |
| #7 | #5 OR #6 |
| #6 | Tongmai hypoglycemic capsule' |
| #5 | Tongmai Jiangtang' |
| #4 | #1 OR #2 OR #3 |
| #3 | Diabetic polyneuropathy ' |
| #2 | Deripheral neuropathy' |
| #1 | Diabetic peripheral neuropathy' |

**Table 4**

The search strategy for Web of Science

| Order | Strategy |
| --- | --- |
| #1 | **(((TS=(**diabetic peripheral neuropathy**)) OR TS=(**peripheral neuropathy**)) OR TS=(**diabetic polyneuropathy**)) OR TS=(**DPN**)** |
| #2 | **(TS=(**Tongmai Jiangtang**)) OR TS=(**Tongmai hypoglycemic capsule**)** |
| #3 | **((((TS=(random)) OR TS=(randomization)) OR TS=(randomized)) OR TS=(randomised)) OR TS=(randomly)** |
| #4 | **#1 AND #2 AND #3** |

**Table 5**

The search strategy for CNKI

| Order | Strategy |
| --- | --- |
| #1 | exp Randomized Controlled trials / all subheadings |
| #2 | Random* |
| #3 | #1 **OR** #2 |
| #4 | exp Tongmai Jiangtang capsule/ all subheadings |
| #5 | diabetic neuropathy |
| #6 | diabetic polyneuropathy |
| #7 | diabetic peripheral neuropathy |
| #8 | #5 **OR** #6 **OR** #7 **OR** #8 |
| #9 | Human |
| #10 | **#3 AND #4 AND #8 AND #9** |

All of the search terms were translated to Chinese terms when we conducted the searches in CNKI database.

**Table 6**

The search strategy for Wangfang Data

| **Title/Topic/Keyword=( "diabetic peripheral neuropathy" or "diabetic neuropathy" or "diabetic polyneuropathy") AND Title/Topic/Keyword=("**Tongmai Jiangtang***")** |
| --- |

All of the search terms were translated to Chinese terms when we conducted the searches.

**Table 7**

The search strategy for VIP

| **Title/Topic/Keyword=( "diabetic peripheral neuropathy" or "diabetic neuropathy" or "diabetic polyneuropathy") AND Title/Topic/Keyword=("**Tongmai Jiangtang***")** |
| --- |

All of the search terms were translated to Chinese terms when we conducted the searches in VIP database.

**Table 8**

The search strategy for Sinomed

| Order | Strategy |
| --- | --- |
| #1 | exp Randomized Controlled trials / all subheadings |
| #2 | Random* |
| #3 | #1 **OR** #2 |
| #4 | exp Tongmai Jiangtang capsule/ all subheadings |
| #5 | diabetic neuropathy |
| #6 | diabetic polyneuropathy |
| #7 | diabetic peripheral neuropathy |
| #8 | #5 **OR** #6 **OR** #7 **OR** #8 |
| #9 | Human |
| #10 | **#3 AND #4 AND #8 AND #9** |

All of the search terms were translated to Chinese terms when we conducted the searches in CBM database.

**Table 9**

The search strategy for Registers (ClinicalTrials.gov, http://www.chictr.org.cn/)

| **Title/Topic/Keyword=("**Tongmai Jiangtang***")** |
| --- |

All of the search terms were translated to Chinese terms when we conducted the searches in http://www.chictr.org.cn/.
